# The impact of SARS-COV2 infection on people in residential care with Parkinson Disease or parkinsonisms: Clinical case series study

**DOI:** 10.1371/journal.pone.0251313

**Published:** 2021-05-06

**Authors:** Chiara Sorbera, Amelia Brigandì, Vincenzo Cimino, Lilla Bonanno, Rosella Ciurleo, Placido Bramanti, Giuseppe Di Lorenzo, Silvia Marino

**Affiliations:** IRCCS Centro Neurolesi “Bonino Pulejo”, Messina, Italy; Hudson Institute, AUSTRALIA

## Abstract

On March 2019 the World Health Organization declared Coronavirus disease (COVID-19) pandemic. Several recent reports disclose that the outcome of the infection is related to age, sex and can be influenced by underlying clinical conditions. Parkinson’s disease (PD) and other parkinsonisms are the most common chronic disease which can cause, directly or indirectly, the patient to be more exposed to other diseases, mostly respiratory system’s ones. Our primary outcome is to evaluate if PD patients are more susceptible than non-PD to take COVID-19 infection. Second, to detect if the infection course is worse in PD-COVID+ patients versus non-PD. This is a retrospective observational study on a cohort of 18 patients (13 PD– 5 non-PD), hospitalized in a Rehabilitative Unit during the occurrence of SARS-CoV2 epidemic outbreak. All patients performed laboratory tests, lung Computed Tomography (CT) and have been tested for COVID-19 thorough pharyngeal swab. PD and non-PD groups were comparable for age, gender and Hoehn and Yahr stage. Seventy-seven (77)% of PD and 60% of non-PD resulted positive for COVID-19. PD-COVID+ and PD-COVID- did not differ for age, disease duration and L-dopa daily dose. PD COVID-19+ subjects were mainly asymptomatic (50%) while non-PD ones were all symptomatic, mostly with respiratory difficulties. PD doesn’t seem to be a risk factor to take SARS-COV2 infection, even if our study is related to a limited sample size. Our results, together with those of other recent studies, highlight the need to evaluate the actual susceptibility of patients with Parkinson’s disease to develop COVID-19 disease, and how the infection may influence the risk of clinical worsening and increase of mortality.

## 1. Introduction

The World Health Organization (WHO) declared the severe acute respiratory syndrome coronavirus 2 (SARS-CoV-2) outbreak a pandemic due to the dramatic increasing of case number outside China [1, 2], on March 11, 2020.

Recent studies on SARS-CoV-2 showed that the infection can affect directly the brain, including brainstem, in both experimental animals and patients [3]. In experimental models, some authors used intranasal virus inoculation to clarify the way in which SARS‐CoV-2 entered the Central Nervous System (CNS), via the olfactory nerves [3]. Interestingly, they found that virus was located in brain, but not in the lung, suggesting a direct CNS transfer via olfactory nerves [4]. In addition, however, the potential involvement of vagus nerve, ambiguus and solitary tract nuclei, which are strictly correlated to brainstem, could be responsible for the infection spreading to CNS from the respiratory tract. In this sense, it’s very relevant the role of cardiorespiratory center, located in the brainstem, for the severe respiratory distress caused by COVID19 [5].

Several studies have described typical COVID-19 clinical manifestations including fever, cough, anosmia, ageusia, diarrhea, fatigue and other symptoms. COVID-19 also presented characteristic laboratory findings and chest computed tomography (CT) abnormalities.

Although the risk factors of COVID-19 remain unclear, many studies reported that male sex represented more than half of the cases. In addition, a large sample of patients presented underlying conditions [6]. For patients with SARS-CoV2 pneumonia, Chen et al. showed that 50.5% of patients had chronic medical illness such as cardiovascular and cerebrovascular diseases (40.4%). In summary, a lot of studies reported that a) most of COVID-19 patients are elderly patients and middle-aged adults, b) there is a higher prevalence of males compared to females, c) severe cases are more likely to have comorbidities (hypertension, followed by diabetes mellitus) than non-severe cases [7, 8].

The increased vulnerability of patients affected by chronic disease, such as Parkinson’s disease (PD) or other parkinsonisms, should be considered very relevant. According to literature, it is well known that elderly subjects with underlying chronic disease have a greater risk to develop a severe disease, or even death. PD is one of the most common age-dependent degenerative disorder and is often associated with comorbidities, particularly cardiovascular diseases [9]; for this reason, PD patients will almost certainly fall into the high-risk group to take SARS COV 2 infection. In addition, there is also an “indirect risk” related to the reported correlation between PD, age and cardiovascular comorbidities [10]. Last, there is another direct risk for a major severity of COVID-19 disease obviously related to PD patients with a pre-existing respiratory dysfunction. In advanced stage of PD, respiratory muscle weakness and abnormal posture determining respiratory muscle rigidity and inadequate respiratory excursions, lead to ventilator failure [11, 12]. Pneumonia was identified as the most frequent reason for hospital admissions and as the most common cause of mortality for PD patients [11, 13]. Other studies also demonstrated that PD patients have a higher probability of dying from pneumonia [14].

To date, no sufficient data are reported to show if PD could increase the risk for COVID-19 infection [15]. Although, looking at the experience from the three most heavily affected regions in Italy [16] and according to preliminary analysis of Italian Institute of Health, it would not seem that Parkinson’s disease is associated with a major risk to contract SARS-COV-2 infection or a to lead to a worse prognosis.

To date, no data are available about the impact of the SARS-COV2 infection on PD patients’ survival in a Movement Disorder Rehabilitative Unit. We performed a descriptive report of our PD population during COVID-19 pandemic, to evaluate the susceptibility of patients with Parkinson’s disease and parkinsonisms to develop the disease, and how the infection may influence the risk of clinical worsening and increase of mortality.

## 2. Materials and methods

### 2.1 Study population

This is a retrospective, observational study. All patients were assessed from 15 February to 26 March 2020, in Movement Disorder Rehabilitative Unit of IRCCS Centro Neurolesi "Bonino-Pulejo" of Messina (Italy).

We enrolled 18 patients (mean age 65 ± 11.02). All patients have been admitted 30 ± 17.3 days (range 7–60) before the occurrence of SARS-CoV2 epidemic outbreak.

Among them, 13 patients (8 males, 5 females) were affected by PD (10 Idiopathic PD, 1 Vascular Parkinsonism (VP), 1 Progressive Supranuclear Palsy (PSP)-like Parkinsonism, 1 Glucosidase Beta Acid (GBA)-associated Parkinson’s disease) and 5 (4 males, 1 female) suffered from other neurological diseases. We use in the text the term PD-group to refer to all parkinsonian patients, including parkinsonisms, since the sample was rather small and the patients had similar demographic characteristics, disease duration, clinical picture and treatment. All patients were hospitalized to perform rehabilitative treatment. Patients performed a multidisciplinary aerobic, motor-cognitive and intensive rehabilitative treatment training (MIRT) specifically designed for patients with PD [17]. MIRT is applicable in any stage of the disease, according to half-yearly cycles, showing more efficacy on progression of disability in early stage of the disease [18].

A confirmed case of COVID-19 was defined as a positive result on high-throughput sequencing or real-time reverse-transcription polymerase chain reaction analysis of throat swab specimens. We performed lung Computed Tomography (CT) and laboratory exams. The infection spread in our Unit in few days without identification of a clear and definite first positive patient. All symptomatic patients, have interrupted rehabilitative treatment, in order to be transferred in specific COVID-Hospital. However, no one have had any worsening in motor or cognitive performance as assessed in first, third and fifth day after infection and through phone call in the next weeks.

### 2.2 Standard protocol approvals, registrations, and patient consents

No ethical committee approval was necessary according to national regulations because this was a retrospective analysis of routinely collected anonymized clinical data. However, the written informed consent was obtained from all patients or legal guardian. A descriptive analysis was performed to illustrate the groups.

## 3. Results

We found SARS-CoV2 RNA positivity in 77% of PD-group (n = 10) and 60% of non-PD group patients (n = 3), while 23% (n = 3) of PD and 40% (n = 2) of non-PD patients resulted negative.

In PD group average age was 68.1±7.80 years (range 58–83 years) with disease duration of 9.15±3.87 years (range 3–18 years) (see Table 1). Hoehn and Yahr (H&Y) score was between 2 and 3 for all participants. All PD-patients were treated with levodopa (62% levodopa + carbidopa and 38% levodopa + benserazide) with a daily dose of 553.85±240.19 mg (range 100–950 mg). Twenty-three (23)% of patients (n = 3) had also undergone previous surgical treatments (1 J-PEG for intra-jejunal infusion of levodopa, 1 Subthalamic Nucleus Deep Brain Stimulation (STN-DBS) and 1 right Ventralis Intermedius Nucleus Magnetic Resonance Guided Focused Ultrasound Surgery (VIM MRg-FUS). Thirty-eight (38)% of population (n = 5) was treated with dopamine agonists (60% pramipexole, 40% ropinirole), 8% (n = 1) with rasagiline + tolcapone, 8% (n = 1) with rasagiline + opicapone, 8% (n = 1) with safinamide + pramipexole. All patients were not dysphagic. In non-PD group, the average age was 57.8± 16.39 (range 38–75). Their neurological disorders were: 20% (n = 1) mild cognitive impairment (MCI), 20% (n = 1) subacute combined degeneration of spinal cord, 20% (n = 1) stroke, 20% (n = 1) Neurodegeneration with Brain Iron Accumulation (NBIA), 20% (n = 1) dorsal myelopathy plus myasthenia gravis (MG) (see Table 1). All patients were not dysphagic.

**Table 1:** Demographic and clinical characteristics of PD and No-PD groups.

	PD	No PD
**Number of patients**	13	5
**Age (Median (I-III quartile)**	67.0 (62.0–76.0)	55.0 (47.0–74.0)
**Gender**		
Male	8	4
Female	5	1
**Disease Duration (Median (I-III quartile) years**	9.0 (7.0–11.0)	1.0 (0.69–8.0)
**H&Y (Median (I-III quartile)–evaluated only on PD patients-**	2.0 (2.0–3.0)	-
**Medical treatment (frequency)**	62% Levodopa+Carbidopa	20% BDZ
		40% LMWH
	38% Levodopa+Benserazide	20% Vit. B12
		20% Iron Chelator

^×^Chi-square test.

^±^Mann-Whitney U test.

PD = Parkinson Disease; SD = Standard Deviation; H&Y = Hoehn and Yahr scale; BDZ = Benzodiazepines; LMWZ = Low molecular weight heparin.

### 3.1 PD-COVID+ group

This group was composed by 10 patients (6 males, 4 females). Among them, 8 had Idiopathic PD, 1 VP and 1 GBA-associated Parkinson’s disease. Average age was 67.0±7.36 years (range 58–76 years) with disease duration of 9.6±4.09 years (range 3–18 years). H&Y score was between 2 and 3 for all participants. All PD-patients were treated with levodopa (60% levodopa + carbidopa and 40% levodopa + benserazide) with a daily dose of 530.0± 235.94 mg (range 100–950 mg). Thirty (30)% (n = 3) have previously undergone advanced treatments (1 J-PEG for intra-jejunal infusion of levodopa, 1 for STN-DBS and 1 for right VIM MRg-FUS treatment). Fifty (50)% of the population (n = 5) was treated with dopamine agonists (60% pramipexole, 40% ropinirole), 10% (n = 1) with rasagiline + tolcapone, 10% (n = 1) with rasagiline + opicapone (see Tables 1 and 2).

**Table 2:** Demographic and clinical characteristics of 4 groups (PD Covid+, PD Covid-, No PD Covid+, No PD Covid-).

	PD COVID+	PD COVID-	No PD COVID+	No PD COVID-
**N. patients**	10	3	3	2
**Age (Median)**	67.0	66.0	74.0	42.5
**(I-III quartile)**	(60.5–74.5)	(66.0–74.5)	(64.5–74.5)	(40.2–44.7)
**Gender**				
Male	6	2	3	1
Female	4	1	0	1
**H&Y (Median) (I-III quartile)–evaluated only on PD patients-**	2.0 (2.0–2.75)	2.0 (2.0–2.5)	-	-
**Levodopa dose (mg) (Mean±SD)**	575.0 (512.5–600)	800.0 (550.0–800.0)	-	-
**Main comorbidities (presenting in more than 2 patients)**	Osteoporosis -Hypovitaminosis D- Hypertension- Diabetes type II- Chronic Venous Insuffency (CVI)	Previous ab ingestis pneumonia, Hypertension	Hypertension–Atrial fibrillation	-

*P<0.05.

^+^ Kruskal-Wallis test.

^×^Chi-square test.

^±^Mann-Whitney test.

PD = Parkinson Disease; SD = Standard Deviation.

### 3.2 PD-COVID—group

This group was composed by 3 PD-patients (2 males, 1 female), 67% (n = 2) affected by Idiopathic PD and 33% (n = 1) by PSP-like Parkinsonism. Average age was 71.66± 9.81 (range 66–83 years); disease duration was 7.66± 3.21 years (range 4–10 years). H&Y score was between 2 and 3 for all participants. All patients took levodopa (67% + carbidopa, 33% + benserazide) with an average daily dose of 633.33±288.67 mg (range 300–800 mg). One patient also took safinamide and pramipexole (see Tables 1 and 2). No advanced treatments were reported.

### 3.3 nonPD-COVID+ group

These patients were 3 males, affected by MCI, previous stroke and subacute combined degeneration of spinal cord, respectively. Average age was 68.0 ± 11.27 years (range 55–75 years) (see Tables 1 and 2).

### 3.4 nonPD-COVID- group

Two non-PD patients (1 male and 1 female), suffering from NBIA and dorsal myelopathy plus MG, respectively, showed an average age of 42.5 ± 6.36 years. They did not presented comorbidities (see Tables 1 and 2).

### 3.5 COVID-19 clinical features

#### 3.5.1 PD-group

As regards the clinical onset and subsequent course, 50% (n = 5, 3 females and 2 males) were completely asymptomatic. They were all affected by Idiopathic PD; 1 of them had undergone right VIM Mrg-FUS treatment few months before. Average age was 69.4±7.46 years (range 58–76 years) with a disease duration of 8.0±1.58 years (range 6–10). They all took levodopa (60% + benserazide, 40% + carbidopa); 60% (n = 3) assumed also pramipexolo and 1 patient ropinirole. 80% (n = 4) suffered from hypertension, 60% (n = 3) from diabetes type II.

Forty (40)% of PD COVID+ patients (n = 4) showed mild symptoms of the infection such as intermittent fever (< 38°), myalgia, phariyngitis. Among these, 1 female was affected by VP, 2 males by Idiopatic PD and 1 male by GBA-associated Parkinson’s disease. One of these also had undergone STN-DBS several years before. Fifty (50)% of these patients showed mild symptoms and lung CT findings of pneumonia. The average age of this group was 64.0±1.58 years (range 58–76 years) with a mean disease duration of 11.0±6.16 years (range 3–18 years). In addition, 80% suffered from hypertension (n = 3), 1 patient from obesity.

Only one patient presented more relevant symptoms with fever (T° > 38.5), interstitial pneumonia with respiratory difficulties that required oxygen-therapy. He was a 67 years old male, with a disease duration of 12 years, in treatment with intrajejunal infusion of levodopa and ropinirole. He had no other comorbidities unless osteoporosis and mild hypovitaminosis D.

#### 3.5.2 Non-PD group

All 3 males non PD-patients that resulted positive to SARS-CoV2, showed symptoms. One of them reported mild symptoms: intermittent fever (T° < 38) without radiological findings of pneumonia. He was a MCI 75 years old subject, with gait impairment due to a previous hip fracture and he suffered also from atrial fibrillation, hypertension. Sixty-seven (67)% of this group (n = 2) was composed by one stroke male of 55 years old, without comorbidities and one male of 74 years old with chronic kidney disease (CKD) and hypertension. They both reported relevant high fever (T° > 38,5), radiological findings of interstitial pneumonia, dispnea and reduction of blood oxygenlevel detected through Arterial Blood Gas (ABG) test. They also needed to be treated with oxygentherapy.

## 4. Discussion

To our knowledge, this is the first report of a cohort of PD patients with SARS-COV2 hospitalized in a Rehabilitative Unit. This provided us a unique opportunity to test the relationship between PD and Covid-19 infection.

Our primary outcome was to analyze if PD could be considered as a risk factor for the infection in terms of susceptibility, specifying for the involvement of the respiratory tract, often favored by bradykinesia. We found 13/18 patients positive for COVID-19; among these, 10 suffered from PD and 3 by other neurological disease. Despite the small number of the nonPD-COVID+ group, due to the fact that our Unit is mainly addressed to patients suffering from movement disorders, we found similar percentage of positive patients in both group (PD-COVID+ group vs nonPD-COVID+ group). For this reason, we can suppose that PD could be not considered as a risk factor for SARS-COV2 infection and that the risk is probably comparable to the elderly general population. Moreover, no significative differences were found between the PD-COVID+ and PD-COVID- negative patients as regards age, L-Dopa daily dose and disease duration. Two patients of PD-COVID+ group took also rasagiline, one patient tolcapone and one opicapone. None of the PD-Covid- group took rasagiline and catechol-O-methyltransferase (COMT) inhibitors, but one took safinamide. According to these data, we can suppose, even if indirectly, the absence of potential susceptibility to the infection of the Parkinsonian patients as it is not linked to any specific factor. In addition, all patients were not dysphagic. This is an important factor, because it is known that advanced PD stage is often complicated by the risk of aspiration pneumonia as a consequence of dysphagia [19].

As a secondary outcome, we tried to identify if there were significative differences as regards the course of the infection among PD-COVID+ and nonPD-COVID+ group. Although we did not find statistically significant results, our data showed that PD COVID-19 positive subjects were mainly asymptomatic (50%) or presented mild clinical features (fever, myalgia, pharyngitis). Just one patient presented more relevant symptoms with high fever, dyspnea and interstitial pneumonia. He was an advanced PD male, with a disease duration of 12 years, in treatment with intrajejunal infusion of levodopa and ropinirole, without other comorbidities unless osteoporosis and mild hypovitaminosis D. Non-PD COVID-19 positive subjects were all symptomatic, presented with fever and more than 50% had respiratory difficulties with radiological findings of interstitial pneumonia. Indeed, no statistical confirm has reported, because of the small sample population.

Patients have been observed for a maximum of 7 days after contracting the infection in our Unit, since they were transferred, as per the regional procedure, to the Reference Center for the treatment of the New Coronavirus disease.

Our findings seem to report that PD shouldn’t be considered as a risk factor for SARS-COV2 infection, even if our sample demonstrated clearly the vulnerability of Rehabilitative Unit that allowed the spread of the infection throughout the presence of common spaces in which patients worked-out daily, although personal protective equipment were used by all staff and other resident patients, as strongly recommended by Italian Health Minister and WHO.

Moreover, from our results, the infection course did not seem to be worse in PD-COVID+ patients versus nonPD, even if this group was composed by a very small number of subjects. In addition, comorbidities seem do not have a predictive role, but this particular variable needs to be described in further studies.

This study itself has, however, several limitations. First of all, the few cohort and control patients’ number that could not have statistically significant results, even if this is the first study on PD SARS-COV2 infected population during rehabilitative hospitalization. Second, during the outbreak period of COVID-19, neuroimaging evaluation, including the possibility to perform magnetic resonance imaging for research studies, was avoided in order to reduce the risk of cross infection. Thus, we do not have neuroimaging data about neurological outcomes for these patients. We would have liked to analyze also the olfactory system as it is known the presence of smell impairment in PD population [20]. In addition, recent studies showed brainstem alteration after SARS-COV2 infection [5] and MRI reports regarding basal ganglia involvement in COVID+ PD patients should have been very interesting. Differently to other experience, in our series no one patient died or experienced a motor and non-motor worsening, as well as no one needed invasive ventilation except for one who needed oxygentherapy [21]. Lower mean age and disease duration, which are considered as risk factors for worse clinical features and mortality in SARS-CoV-2 infection [21, 22], probably explain why in our PD-COVID+ group better evolution was observed. Moreover, no one required any increase in levodopa dosage.

A very recent study seems to confirm our findings, supposing that COVID-19 risk and mortality did not differ from the general population [23]. Conversely, Cilia et al. [24] reported that motor and nonmotor symptoms significantly worsened in PD COVID-19 patients, even if the data were related to only 12 COVID-19 cases.

Another study reported an increased case fatality rates (CFR) in patients with PD, not related to age, sex, and race, even if authors were unable to account for confounding regional factors that could increase mortality and “key-comorbidities” were not reported [25]. Kobylecki et al. [26], highlighted the vulnerability of patients with PD in hospitals and in the community during COVID-19 pandemic. Fasano et al. [27] underlined how the fragility caused by advanced PD poses an increased risk of mortality during COVID-19.

In conclusion, the coexistence of PD diagnosis was not found to be a primary risk factor for SARS-COV2 infection, neither increased the susceptibility, even if, especially advanced PD, COVID-19 disease, could worse motor and nonmotor symptoms and increase risk of mortality, as other studies reportes [24–27].

In case of SARS-COV2 infection, it is mandatory to maintain previous PD medications, especially the adequate and recommended dosages of L-Dopa/DA. In this way, we can avoid respiratory impairment due to bradykinesia and rigidity that could reduce vital capacity and peak expiratory flow.

Our results, together with those of other recent studies, place the need to evaluate the actual susceptibility of patients with Parkinson’s disease to develop the COVID-19 disease, and how the infection may influence the risk of clinical worsening and increase of mortality.

## Supporting information

S1 Database (XLSX)
